# The Relationship between Personality Styles of Sociotropy and Autonomy With Suicidal Tendency in Medical Students

**DOI:** 10.5539/gjhs.v7n3p345

**Published:** 2015-03-10

**Authors:** Ahmadali Raeisei, Azizollah Mojahed, Nour-Mohammad Bakhshani

**Affiliations:** 1 Health Promotion Research Center, Zahedan University of Medical Sciences, Zahedan, IR Iran; 2 Children and Adolescents’ Health Research Center, Zahedan University of Medical Sciences, Zahedan, IR Iran

**Keywords:** sociotropy, autonomy, suicidal tendencies, medical students

## Abstract

The research aim was investigating the relationship between personality styles of autonomy and sociotropy, and suicidal behavior at Zahedan University of medical sciences’ medical students. This was a descriptive correlational study. The population consisted of all medical students at Zahedan University of Medical Sciences internship period 2002-2003. The number of samples was 102 patients, including 47 males and 55 females. To collect information, the personal style inventory (PSI) with 48 items. Twenty four items to assess sociotropy, 24 items to assess autonomy, and to measure suicide the suicidal subscale (MMPI) with 21 items were used. The two scales had the content validity and for the reliability used Cronbach α. So the reliability of the personality styles is 0.84 and the reliability of the suicidal subscales is 0.83. Data were analyzed using Pearson’s correlation methods. The results showed that there is an inverse and significant relation between autonomic style and trends of suicide in men (*P* = 0.02, *r* = -0.43), but no association between sociotropy and suicidal tendencies were observed in men. There was no significant relationship between autonomy and sociotropy personality styles and tendency towards suicide in women.

## 1. Introduction

Suicide is one the eight leading cause of death in most countries and victimize one million people in the world annually. It is said that the real numbers, including attempted suicide and suicidal behavior is 10 to 20 times more than this amount (WHO, 2004). Suicide and attempted suicide is one the most important indicators of mental health of the population which result from the effect of various factors such as age, sex, marital status, economic situation, family, drug abuse, struggle between parents and Divorce, lack of social support, unemployment, physical and mental illness, particularly depression and suicidal thoughts and suicide attempts are all relevant factors to suicidal attempts.

Regarding the etiology of suicide and attempted suicide several factors have been identified in which the results of the studies showed that in more than 90% of suicides, psychiatric disorders (Robins, Murphy, Wilkinson, Gesner & Keys 1959; Dorpet and Ripley 1960; Barraclough, Bunch, Nelson, & Sainsbury, 1974) have key roles. Among mental disorders, mood disorders held the highest rate of suicide in both sexes (Roy, 2004). Mental disorders, especially bipolar disorders play an important role in suicidal behavior. Currently, it is estimated that more than 30 percent of people who lose their lives due to suicide, had personality disorders (Oldham, 2006). It seems that personality disorders besides suicide have other effects which in turn affect the suicide such as the loss of social support, negative repeated life events, and poor health care. Also, it seems that bipolar disorders are related more to the impulsivity and aggressive behavior (Nabuco et al., 2009). In this field, Researchers like Leung, Lai, Yu & Fu (2012), Segal et al., (2012), Black et al.(2004), Sharp et al.(2012) and Soltaninejad et al.(2013) concluded that the borderline personality disorders and their characteristics such as hopelessness, impulsivity, emotional instability and interpersonal turmoil are significantly related to suicide and suicidal ideation and non-suicidal self-injury behavior. Among psychiatric disorders, depression with 58% has allocated the highest rates of suicide risk (Zare & Sayadi, 2009). All over the world, depression is a serious and disabling health problem with high prevalence rate (Kupfer et al., 2012) and there is a strong relationship between tendencies towards depression and suicide (Handley et al., 2012). Depression is a disorder which is characterized by the reduction of energy and interest, feelings of guilt, difficulty in concentration, poor appetite and suicidal and death thoughts of death and comes along with change in activity level, cognitive ability, speech, sleep, appetite and other biological rhythms (Aylderabadi et al., 2003).

Comparing the mood disorders, with regard to their risks in the lifetime, shows that the lowest risk of suicide (12 %) lies with depression disorder and the highest risk lies with major depression (20%) (Harris & Baraklav, 1998, cited by Ostamo, & Lonnqvist, 2001). Suicide rate in ages of 21 and 22 years is more than suicide rate in individuals younger than 18 or older than 26 years (Panaghy, 2008).

To explain the cognitive pathology of emotional disorders, including suicide in depression, several supportive theories exist which relate the cognitive operation to social thought and behavior and put forward the suicide as either related or sequel to depression since the start of cognitive theory (Beck, 1967). Based on numerous clinical experiences and researches, Beck and his colleagues concluded that, depressed people have negative thoughts about themselves, their life experiences and their future, see others rejective and unsupportive and consider themselves, people who have defects in the major aspects. About cognitive characteristics of suicidal people, several theories have been discussed in the field of cognitive pathology theories which each of them has some confirming evidence. Among these concepts, lack of flexibility and black and white thinking are in attempted suicide patients, which have been proposed since the founding of the cognitive models to explain suicide (Ellis & Rutherford, 2008).

Suicidal people feel that their recent problem is unbearable and believe that there is no hope for change. Therefore, negativism and hopelessness about the future, as well as a pessimistic view about themselves, the world, and others are important factors that lead to commit suicide a person who is depressed and the outcome of this type of thinking is the suicide as the solution which comes to their mind (Reinecke, Dubios, & Schulz, 2001).

In a study that Hübner et al. (2010) was done on the reduction of suicide through the alliance against depression in Germany, after five years of intervention therapy in depressed patients and conducting the research, the suicide rate was measured before and after intervention. The result showed that the suicidal rate was reduced significantly during the intervention period, especially due to the significant reduction of suicides among men. This study is carried out to investigate the relationship between personality styles of autonomy and sociotropy with suicidal behavior in medical students and is intended to investigate the following questions:

Is there a relationship between sociotropy personality style and suicide intention among male and female medical students?

Is there a relationship between autonomy personality style and suicide intention among male and female medical students?

Is there a relationship between subscale personality styles of sociotropy and autonomy with suicide intention among medical students with respect to sex?

## 2. Methodology

The method of this descriptive research was descriptive-correlational. The population consisted of all medical students at Zahedan University of Medical Sciences internship during period 2002-2003 academic year. The samples were 102, including 47 males and 55 females. To collect information, the personality style test questionnaire (PSI) with 48 items, 24 items to assess sociotropy, 24 items to assess autonomy, each of these two characters had three minor factors with three sub-factors: 1). Being worry about what others think. 2). Dependency. 3). to satisfy others related to sociotropy and three sub-factors, 1). Perfectionism / self-criticism, 2). The need for control, and 3) the Defensive break is concerned about the style of Autonomy and for the assessment of suicide, we used suicidal subscale (MMPI) with 21 items. The questionnaires had the content validity and for the reliability used Cronbach alpha. So the reliability of the personality styles was 0.84 and the reliability of the suicidal subscales was 0.83. After the data were collected, they were coded and entered into the computer using the statistical software SPSS. 16, and the descriptive and inferential statistical analysis were performed.

**Ethical Consideration**

The Committee of Zahedan University of Medical Sciences approved this study. This research is extracted from M.D thesis with allocated ethical code from Committee of Ethics. This study has conducted according to the principles expressed in the Declaration of Helsinki. A letter of permission to conduct the study was taken from the School of Medicine of Zahedan University of Medical Sciences to seek permission from authorities. Ethical Clearance for the study was also obtained from the Zahedan University of Medical Sciences Ethical Review Board.

## 3. Research Findings

The study sample comprised of all medical interns who passed internship wards during 2003, including 47 males and 55 females. Ladies were 53.9% and gentlemen 41.1% of this study population. Average score of students’ sociotropy was 95.07 with standard deviation of 17.49 and students’ autonomy score was 90.58 with a standard deviation of 14.61. The average score of sociotropy in men was 87.5 with a standard deviation of 16.8 and in autonomy 84.30 with a standard deviation of 11.33. In women, the average score of sociotropy was 101.54 with a standard deviation of 15.43 and in autonomy it was 95.94 with a standard deviation of 15.05.

In men, the mean and standard deviation of sub-personality style of sociotropy “worry about what others think about someone, dependency and satisfying others”, were (22.30, 5.5); (26.63, 6.4) and (38.6, 8.73), respectively and the mean and standard deviation of these styles for women were (28.64, 6.20), (30.65, 6.04) and (42.25, 6.40), respectively.

In men, the mean and standard deviation of sub-personality style of autonomy “perfectionism, self-criticism, the need to defensive control and break “, were (13.30, 3.40), (28.6, 5.25) and (42.40, 6.38), respectively and in the case of women, the mean and standard deviation of the same sub-styles were (17.90, 6.81), (32, 6.10) and (45.07, 8.37), respectively. Mean and standard deviation scores of the personality styles and sub-styles are shown in Tables 1 and 2.

**Table 1:** Mean and standard deviation of sociotropy personality style with respect to sex

Sociotropy Sub-scales								

	Being worry about what people think of me		Dependency		Satisfying others		Sociotropy	
	Mean	SD	Mean	SD	Mean	SD	Mean	SD
Men	22.30	5.5	26.63	6.4	38.6	8.73	87.5	16.8
Women	28.64	6.20	30.65	6.04	42.25	6.40	101.54	15.43

**Table 2:** Mean and standard deviation of Autonomy personality style with respect to sex

Autonomy Sub-scales								

	Perfectionism, Self-criticism		Needs to control		Defensive break		Autonomy	
	Mean	SD	Mean	SD	Mean	SD	Mean	SD
Men	13.30	3.40	28.6	5.25	43.40	6.38	84.30	11.33
Women	17.90	6.81	32.00	6.10	42.07	8.37	95.94	15.05

Between autonomic personality style and tendency of suicide in men exists an inverse and significant correlation (*P*=0.02, *r*=-0 .43), but no association between sociotropy and suicidal tendencies were observed in men. No significant association was observed between autonomy and sociotropy personality styles and suicidal trends in women. A significant and inverse relationship was observed between sub-scale separation break of autonomy and tendency to suicide in men (P=0.02, *r*=-0. 44). Also, a significant relationship was observed between sub-scale of satisfying others of sociotropy and tendency to suicide in women (*P*=0.035, *r*=-0.29). Significant correlations were not observed between the subscales of “worry about what others think, dependency, and satisfying others” of sociotropy and sub-scales of autonomy personality style “perfectionism-self-criticism, the need to control and defensive break “and suicidal tendency in men.

Significant correlations were not observed between the subscales of “being worry about what others think, dependency, and satisfying others” of sociotropy and sub-scales of autonomy personality style “perfectionism- self-criticism, the need to control and defensive break “and suicidal tendency in women. The relevant data are shown in Table 3.

**Table 3:** The relationship between suicidal tendency and personality styles of sociotropy and autonomy and subscales related to sex in medical interns

		Being worry about others’ thinking	Dependency	Satisfying others	Sociotropy	Perfectionism- Self-criticism	Need to control	Defensive break	Autonomy
Tendency to suicide in men	R	0.2	-0.07	-0.4	0.02	-0.22	-0.28	-0.44	-0.43
	P	0.18	0.65	0.8	0.9	0.15	0.7	0.02	0.02
Tendency to suicide in women	R	0.11	0.14	0.29	0.22	0.06	-0.13	-0.046	-0.05
	P	0.43	0.32	0.035	0.11	0.7	0.36	0.74	0.72

## 4. Discussion and Conclusion

Average score of sociotropy of students was 95.07 with a standard deviation of 17.49, and their autonomy score was 90.58 with a standard deviation of 14.61. The Sociotropy score of women was higher than men because sociotropy is more related to theatrical personality, most women are involved with this disorder and make it justified. The average scores of autonomy and sociotropy in men were 78.5 and 84.30, respectively. In women the scores were 101.54 and 95.94, respectively. In a study conducted in France to evaluate the reliability and validity of the French version of the PSI-II, they studied 202 male and female students. The results demonstrated the three-factor of sociotropy and two-factor for autonomy. There was a significant association between the two sub-styles of sociotropy and two sub-styles of IDI with numbers of 67% (*P* = 0.001) and 44% (*P* = 0.001), and second between sub-styles of autonomy PSI with the numbers 39% (*P* = 0.001), respectively. The sociotropy numbers were 93.98 (SD = 14.22) and 88.55 (SD = 11.69), respectively. These results show that autonomy is more in Iranians.

In general, the sociotropy scores were higher than autonomy scores. There was a significant association between autonomy and sub-scale of defensive break and suicidal tendency in men and in women. There was a significant association between sub-scales of satisfying others with suicidal tendency.

In the present study, a significant and inverse relationship exists between autonomy and suicidal tendencies in men. That means the more the autonomy the less is likely to be suicide. The less likelihood of suicide might be due to the fact that these people feel more independent in their life and have more self-confidence. However, in some researches the results were different and suicidal tendency was more. In a study on people who had neuroticism despair, introversion, and feeling controlled by others- more characteristic of autonomy personality- had more suicides (Annette et al., 1999). Also, in another study, it was shown that autonomy is likely to cause depression and subsequent suicidal behavior, due to lower social support and more interpersonal problems (Cample et al., 2003). Among women, no correlation was observed between autonomy and suicidal tendencies, which might be due to above-mentioned reasons. In highly sociotrope participants the regression analysis was significant and also negative life events and social support were identified as significant predictors; in contrast with, in highly autonom participants the regression analysis was significant (F (2, 15) = 6.33, p<0.01) but unique contribution of social support was not Significant (Bakhshani, 2007).

The findings of the study showed that in men and women there is no significant relationship between personality style of sociotropy and suicidal tendency. It is likely because of the more interpersonal and social relationship and more supportive expectation from others, while in a study, the patients with high sociotropy have reported more negative interpersonal events than depressed patients with high autonomy (C. J. Robins, 1990). The findings of the research have also showed that in men, there is a significant and inverse relationship between sub-scale of defensive break of autonomy and suicidal tendency, i.e., the more defensive break, the less tendency towards suicide. Perhaps this is also because of sense of independence and self-confidence in them. Among women, there was a significant relationship between the sub-style of satisfying others and suicidal tendency. Perhaps, this relationship is due to the conflicts between their attempts to satisfying others and getting negative answers from them and not meeting their expectations from interpersonal and other relationships.
